# Spinal sagittal alignment and trapezoidal deformity in patients with degenerative cervical spondylolisthesis

**DOI:** 10.1038/s41598-019-41079-3

**Published:** 2019-03-21

**Authors:** Kazuma Murata, Kenji Endo, Hidekazu Suzuki, Yuji Matsuoka, Taichiro Takamatsu, Hirosuke Nishimura, Kengo Yamamoto

**Affiliations:** Department of Orthopedic Surgery, Tokyo Medical University, 6-7-1 Nishishinjuku, Shinjuku-ku, Tokyo Japan

## Abstract

Degenerative cervical spondylolisthesis (DCS) is a cervical deformity arising from regressive changes where trapezoidal deformity characterized by hypertrophic osteophytes of spinal elements is often observed. There is a paucity of literature about the spinal contour of DCS and trapezoidal vertebrae. We conducted this research to clarify the relationship between spinal sagittal alignment and trapezoidal deformity in DCS. Total seventy-nine patients with cervical spondylosis were enrolled. Twenty-four patients who exhibited cervical spondylolisthesis were classified into DCS group. Other patients were classified into a control group. Measurements of radiographic parameters and trapezoidal deformity were made. DCS was found mostly in C3-C4 and C4-C5 (16 and 10 cases, respectively). T1S and T1-T4 TK was larger in the DCS group than in the control (T1S: 29.9 ± 2.3° vs. 23.7 ± 1.5°, T1-T4 TK: 14.9 ± 2.1° vs. 9.0 ± 1.4°). C2-C7A was smaller in DCS (3.5 ± 3.6° vs. 11.9 ± 2.3°). Trapezoidal deformity was apparent in the vertebra below the slipped segment. Among sagittal parameters, T1S and T1-T4 TK were positively correlated with DCS (r = 0.523 and r = 0.438, respectively). For these correlations with DCS, both logistic and linear regression models predicted threshold values of approximately 30° for T1S and 15° for T1-T4 TK responsible for DCS. DCS was mostly found in the middle cervical region. Among sagittal parameters, enlarged T1S and T1-T4 TK, which were strongly correlated with amount of slippage, was considered affected to DCS. Cervical kyphosis and trapezoidal deformity also exhibited strong correlations with DCS, and were considered responsible for clinical instability.

## Introduction

Degenerative cervical spondylolisthesis (DCS) is a form of cervical deformity caused by sliding shear force, and disc degeneration, which is often characterized by hypertrophic osteophytes or spur formation of spinal elements where vertebrae exhibit the trapezoid form^1,2^. Since degenerative cervical spondylolisthesis was first reported in 1986^3^, DCS has received insufficient attention compared to degenerative lumbar spondylolisthesis^4^, perhaps because the incidence of DCS has been considered rare compared to that of degenerative lumbar spondylolisthesis (19.7%), both in the young^5,6^, and in the elderly population^7–9^. DCS, however, may not be so rare, as reported elsewhere^1,2,10–12^. In fact, cervical DCS is considered important as a cause of dynamic canal stenosis and is often overlooked due to unclear compression of the cervical cord in MRI performed in the neutral supine position^13^.

The diagnostic criteria for DCS have not been consistent among reports. In each of several studies, the criteria for diagnosis of DCS were set at 2 or 3 mm^2,10,11^, according to a cadaveric study which demonstrated that no normal adult spine should have horizontal displacement greater than 2.7 mm^14^. From the analysis using kinematic magnetic resonance imaging, DCS may well be considered as unstable if there is greater than 2 mm of horizontal translation where DCS may decrease the space available for the cord in affected segments^15^.

These findings may suggest that DCS is unstable when the amount of slippage is even as small as 2 mm, and is of significance as a compressive element to the spinal cord. Lateral radiographs may be initially performed to detect spondylolisthesis^15^. However, there is a paucity of literature describing the morphologic features of DCS including spinal sagittal alignment and trapezoidal deformity, which is often identified in the segments affected by DCS. The radiographic morphology and the risk factors for DCS are still not well understood. The primary aims of the present study were to clarify the pathophysiology and risk factors for DCS through radiographic measurements.

## Materials and Methods

**Figure 1:**
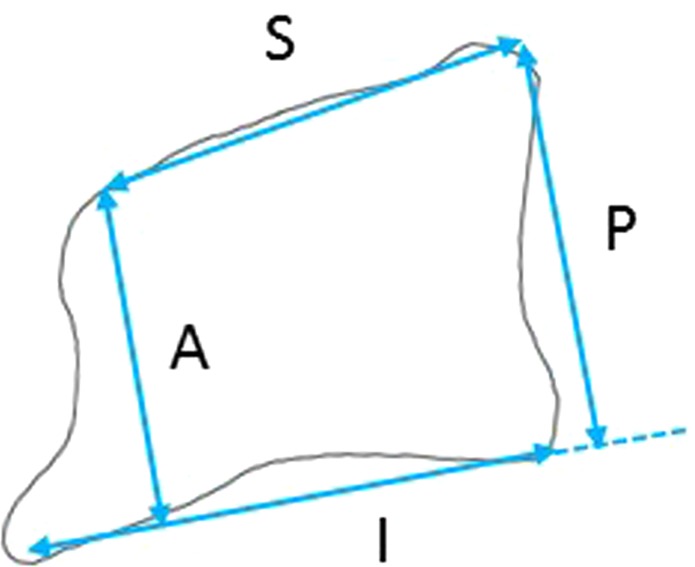
Visual representations of the methods applied to measure trapezoidal features in radiographs are shown as follows: (1) the ratio of anterior wall height to posterior wall height (A/P), (2) the ratio of superior end-plate width to superior end-plate width plus inferior end-plate width (A/SP), and (3) the ratio of superior end-plate width to inferior end-plate width (S/I).

**Figure 2:**
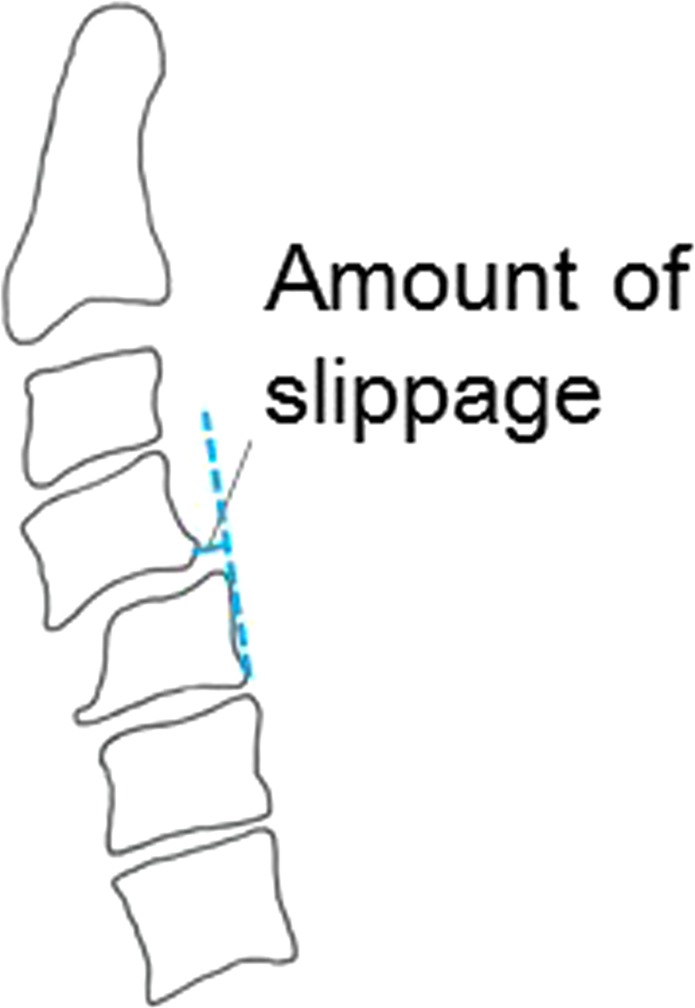
Visual representations of the methods applied to measure amount of slippage in radiographs are shown. Each segmental angulation was measured as the angulation of intersection between lines parallel to the inferior end-plate of the vertebra above and the superior end-plate of the vertebra below.

After obtaining institutional review board approval, a retrospective analysis of radiographic profiles was performed for patients who presented to our department with DCS from 2008 to 2013. All patients received cervical and total spinal lateral radiographs following the standard protocol described below. In a retrospective analysis of consecutive case series of outpatients diagnosed as cervical spondylosis from whom both cervical and total spinal radiographs could be obtained. A total of 79 patients were included. The distribution of symptoms was as follows: myelopathy (n = 29), radiculopathy (n = 8), or neck pain (n = 42). Twenty-four patients who exhibited anterolisthesis of more than 2 mm were classified into DCS group. The remaining 55 patients were included as a control group. The comparison about distribution of symptoms (DCS group: myelopathy n = 8, radiculopathy n = 1, neck pain n = 13; control group: myelopathy n = 21, radiculopathy n = 7, neck pain n = 29, respectively) was not significantly different by Pearson’s chi-square test with Bonferroni correction (p = 0.57). Lateral standing radiographs were obtained by using vertical film with a constant distance between the subject and the radiographic source with a radio-opaque calibration tool, following a standardized protocol with the patients standing in a fist-on-clavicle position and instructed to look straight ahead with knees locked^16^. The following spinal parameters were assessed: (1) C2 slope (C2S), which was measured as the angulation of intersection between lines parallel to the C2 inferior endplate and the horizontal line, (2) C2-C7 lordosis angle (C2-C7A), which was measured as the angulation of intersection between lines parallel to the C2 superior endplate and the C7 inferior endplate, (3) T1 slope (T1S), which was measured as the angulation of intersection between lines parallel to the T1 superior endplate and the horizontal line, (4) C7-S1 sagittal vertical axis (C7-S1SVA), which was measured as the distance between C7 plumb line and S1 supero-posterior corner, (5) T1-T4 thoracic kyphosis angle (T1-T4TK), which was measured as the angulation of intersection between lines parallel to the T1 superior endplate and the T4 inferior endplate, (6) T4-T12 thoracic kyphosis angle (T4-T12TK), which was measured as the angulation of intersection between lines parallel to the T4 superior endplate and the T12 inferior endplate, (7) lumbar lordosis (LL), which was measured as the angulation of intersection between lines parallel to the superior endplate of T12 and the sacrum, (8) sacral slope (SS), which was measured as the angulation of intersection between lines parallel to the superior endplates of sacrum and the horizontal line, (9) pelvic tilt (PT), which was measured as the angulation of intersection between lines parallel to a line connecting the midpoint of superior endplate of sacrum to center of hip axis and the vertical line, and (10) pelvic incidence (PI), which was measured as the angulation of intersection between lines parallel to a line connecting the midpoint of superior endplate of sacrum to center of hip axis and a perpendicular line to the superior endplate of sacrum^17,18^. The accuracy of the methods, including intra-rater and inter-rater agreements were analyzed in detail in a previous paper^19^. We also measured the trapezoidal deformity by the following three methods: (1) the ratio of anterior wall height to posterior wall height (A/P), (2) the ratio of superior end-plate width to the sum of superior end-plate width and inferior end-plate width (A/S + P), and (3) the ratio of superior end-plate width to inferior end-plate width (S/I) (Fig. 1). The slippage was measured by the distance from the posteroinferior corner of the vertebra above to the tangential line along the posterior border of the vertebra below^1^ (Fig. 2). All methods including radiographic analysis and management of private information were performed in accordance with relevant guidelines and regurations.

Statistical analyses were performed using commercially available software (JMP version 11.0, SAS Institute, Inc., Cary, North Carolina, USA). After determining that the data followed a parametric distribution using the Shapiro-Wilk normality test (where *p* > 0.05 suggests that the data are from a normal distribution), Student *t*-tests were calculated to compare the two groups, and Pearson product-moment correlation coefficients were calculated for all combinations of radiographic measurements and amount of slippage, which was considered the most characteristic feature of DCS. The level of significance was set at *p* < 0.05. For significant correlations observed among radiographic parameters, further analysis using both a linear regression and a logistic regression analysis with binary variables were performed to determine a possible threshold of radiographic measurement in which patients with DCS was most significant. Because most previous papers have set at 2 mm the amount of slippage as significant^2,11^, we considered slippage of more than 2 mm as positive. We also included 1–2 mm of mild slippage in statistical analysis as negative in patients to detect optimal thresholds for correlated parameters.

### Ethics Review Committee Statement

This study was approved by the Ethics Review Committee of Tokyo Medical University Hospital. Written informed consent was obtained from all patients before the recruitment of patients in this study.

## Results

### Patient characteristics

Seventeen female and 7 male DCS patients with an average age of 74.3 ± 1.6 years who agreed to participate were enrolled in this study. A retrospective analysis of data showed that the 55 patients in the control group were not significantly different in age (75.4 ± 1.1, *p* = 0.56) and sex ratio (38 female and 17 male patients, *p* = 0.75) from the DCS group. The patients who had evidence of retrograde spondylolisthesis or diffuse idiopathic skeletal hyperostosis inside or outside the cervical region were excluded from both groups.

### Segments revealing DCS

DCS was found in 1 patient at C2-C3 (3.7%), 16 patients at C3-C4 (59%), 10 patients at C4-C5 (37%), and 1 patient at C5-C6 (3.7%); no evidence of spondylolisthesis was found in other segments. Mean value of slippage at each segment was 2.0 mm at C2-C3, 3.9 mm at C3-C4, 3.9 mm at C4-C5, and 3.1 mm at C5-C6, respectively.

### Radiographic measurements

Radiographic measures of the two groups are summarized in Table 1. C2-C7A represented a more decreased cervical lordosis in the DCS group than in the control group (3.5 ± 3.6° vs. 11.9 ± 2.3°, *p* = 0.036). T1S were more significantly larger in the DCS group than in the control (T1S: 29.9 ± 2.3° vs. 23.7 ± 1.5°, *p* = 0.028). T1-T4TK was significantly larger in the DCS group than in the control (14.9 ± 2.1° vs. 9.0 ± 1.4°, *p* = 0.022). In contrast to these differences at the cervicothoracic junction, parameters concerning the thoracolumbar region and global balance seemed not to be different between the two groups.

**Table 1:** Radiographic measures of standing total spine.

	DCS	Control	*p* value
C2S	29.4 ± 4.6°	18.4 ± 3.4°	0.057
C2-C7 A	3.5 ± 3.6°	11.9 ± 2.3°	0.036
T1S	29.9 ± 2.3°	23.7 ± 1.5°	0.028
C7-S1 SVA	20.4 ± 9.0 mm	30.5 ± 5.7 mm	0.346
T1-T4 TK	14.9 ± 2.1°	9.0 ± 1.4°	0.022
T4-T12 TK	28.1 ± 3.8°	30.7 ± 2.2°	0.562
LL	42.5 ± 3.7°	39.4 ± 2.3°	0.492
SS	26.6 ± 2.1°	25.0 ± 1.3°	0.522
PT	21.7 ± 2.5°	27.7 ± 1.6°	0.047
PI	50.0 ± 2.4°	53.1 ± 1.5°	0.270

DCS: degenerative cervical spondylolisthesis.

C2S: C2 slope.

C2-C7 A: C2-C7 lordosis angle.

T1S: T1 slope.

C7-S1 SVA: C7-S1 sagittal vertical axis.

TK: thoracic kyphosis angle.

LL: lumbar lordosis angle.

SS: sacral slope.

PT: pelvic tilt.

PI: pelvic incidence.

### Trapezoidal deformity

Measurements regarding trapezoidal deformity are summarized in Table 2. Trapezoidal change of C3 did not seem different between the two groups. However, those of C4, C5, and C6 were significantly smaller in the DCS group than in the control. These findings might be more apparent in the vertebra below the slipped segment in light of observations at C3-C4 (n = 13) and C4-C5 (n = 10): Mean value of measurement of trapezoidal deformity at C4 was estimated as 0.81 ± 0.02 of C4-A/P (p = 0.001), 0.43 ± 0.01 of C4-A/S + P (p = 0.006), and 0.75 ± 0.03 of C4-S/I in the case with DCS at C3-C4, and trapezoidal deformity of C5 was measured as 0.83 ± 0.02 of C5-A/P (p = 0.001), 0.41 ± 0.01 of C5-A/S + P (p = 0.003), 0.68 ± 0.03 of C5-S/I (p = 0.004) at C4–C5, respectively.

**Table 2:** Measurements regarding trapezoidal deformity.

	DCS	Control	*p* value
C3 A/P	1.02 ± 0.03	1.00 ± 0.02	0.565
A/S + P	0.45 ± 0.01	0.46 ± 0.00	0.143
S/I	0.82 ± 0.02	0.85 ± 0.01	0.144
C4 A/P	0.87 ± 0.02	1.00 ± 0.02	0.001
A/S + P	0.44 ± 0.01	0.46 ± 0.01	0.034
S/I	0.79 ± 0.02	0.86 ± 0.02	0.033
C5 A/P	0.88 ± 0.03	0.96 ± 0.02	0.052
A/S + P	0.42 ± 0.01	0.45 ± 0.01	0.023
S/I	0.74 ± 0.02	0.82 ± 0.02	0.021
C6 A/P	0.98 ± 0.02	1.02 ± 0.02	0.173
A/S + P	0.46 ± 0.01	0.48 ± 0.01	0.027
S/I	0.85 ± 0.03	0.95 ± 0.03	0.025

DCS: degenerative cervical spondylolisthesis.

A/P: the ratio of anterior wall height to posterior wall height.

A/S + P: the ratio of superior end-plate width to the sum of superior end-plate width and inferior end-plate width.

S/I: the ratio of superior end-plate width to inferior end-plate width.

### Statistical analysis

Correlations between DCS and radiographic measurements are summarized in Table 3. These analyses revealed the significant positive and negative correlations between amount of slippage and parameters regarding the cervical region and cervicothoracic junction. In particular, C2-C7A exhibited significant negative correlation with DCS (r = −0.479, *p* = 0.001). T1S exhibited significant positive correlation with DCS (r = 0.523, *p* = 0.001) on the contrary. T1-T4TK exhibited significant positive correlation with DCS also (r = 0.438, *p* = 0.001). Among indexes of trapezoidal deformity at C4 and C5, C4-A/P (r^2^ = 0.33, AUC = 0.86), C4A/S + P (r^2^ = 0.48, AUC = 0.93), C4-S/I (r^2^ = 0.48, AUC = 0.93), C5-A/P (r^2^ = 0.37, AUC = 0.89), C5-A/S + P (r^2^ = 0.27, AUC = 0.83), and C5-S/I (r^2^ = 0.26, AUC = 0.83) exhibited significant conformability in logistic analysis where the evidence of DCS was valued as positive. This logistic analysis revealed the thresholds of DCS for each parameter, and among the indexes of trapezoidal deformity, A/P exhibited relatively consistent thresholds of DCS for C4 and C5.: Estimated thresholds of A/P_below_, A/S + P_below_, and S/I_below_ were 0.90, 0.46, and 0.84 in the case with DCS at C3-C4, 0.90, 0.43, and 0.73 in the case with DCS at C4-C5, respectively.

**Table 3:** Correlations between radiographic measures and amount of DCS.

	Radiographic measures	Correlation (Pearson r)	*p* value
DCS vs.	C2S	0.290	0.010
	C2-C7 A	−0.479	0.001
	C2-C7 SVA	0.356	0.001
	T1S	0.523	0.001
	C7-S1 SVA	−0.116	0.310
	T1-T4 TK	0.438	0.001
	T4-T12 TK	−0.156	0.170
	LL	−0.250	0.027
	SS	−0.112	0.326
	PT	−0.269	0.017
	PI	−0.146	0.199

DCS: degenerative cervical spondylolisthesis.

C2S: C2 slope.

C2-C7 A: C2-C7 lordosis angle.

C2-C7 SVA: C2-C7 sagittal vertical axis.

T1S: T1 slope.

C7-S1 SVA: C7-S1 sagittal vertical axis.

TK: thoracic kyphosis angle

LL: lumbar lordosis angle.

SS: sacral slope.

PT: pelvic tilt.

PI: pelvic incidence.

**Figure 3:**
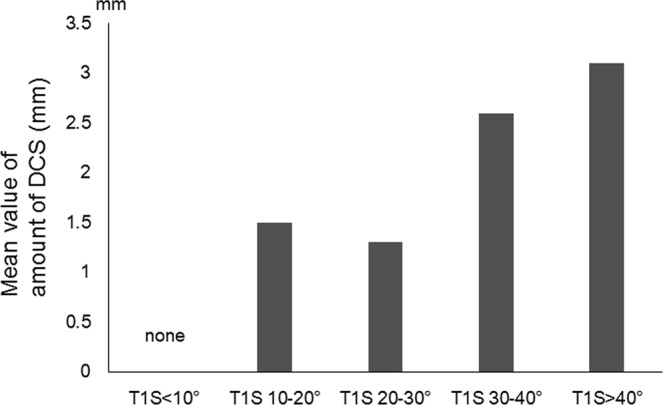
A bar graph of mean data is shown.

**Figure 4:**
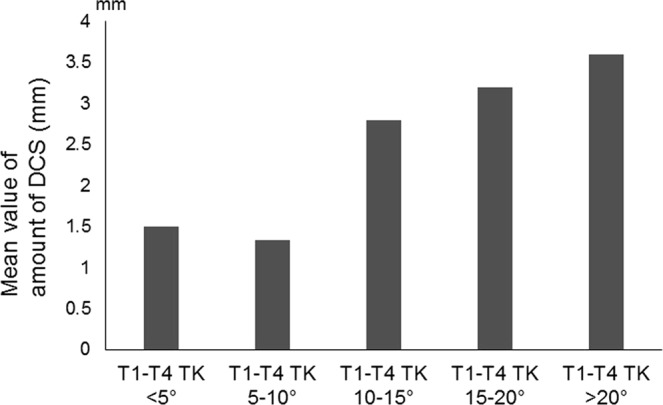
A bar graph of mean data is shown.

**Figure 5:**
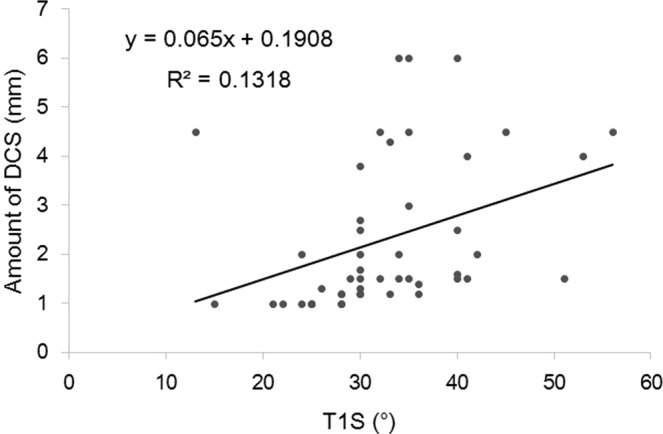
A visual representation of the regression models superimposed on raw data is shown. Positive correlation was observed between T1 slope (T1S) and amount of slippage.

**Figure 6:**
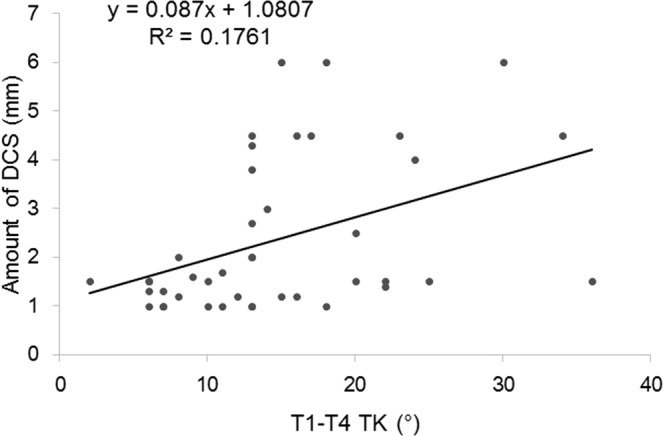
A visual representation of the regression models superimposed on raw data is shown. Positive correlation was observed between T1-T4 thoracic kyphosis angle (T1-T4 TK) and amount of slippage.

Both logistic and linear regression analyses were used to determine a possible radiographic parameter threshold at which DCS was defined by amount of slippage >2 mm. The logistic regression models predicted a value of 30° for T1S (χ^2^ = 40.06, *p* = 0.001) (Fig. 3) and 13° for T1-T4TK (χ^2^ = 21.54, *p* = 0.001) (Fig. 4) at which the *p* value for the correlation tests was lowest. AUC calculated from the ROC curve was estimated at 0.9167 for T1S and 0.7978 for T1-T4TK, which seemed to represent high accuracy of the result. The linear regression models predicted values of 27.8° for T1S (r^2^ = 0.1318, *p* = 0.011) (Fig. 5) and 10.6° for T1-T4TK (r^2^ = 0.1761, *p* = 0.006) (Fig. 6), responsible for cervical positive imbalance. From the logistic regression analysis, relative risk was estimated at 4.2-fold (95% confidence interval [CI] = 2.0–8.9, *p* = 0.001) in the case of T1S > 30°, 2.1-fold (95% CI = 1.1–4.3, *p* = 0.042) in the case of T1-T4TK > 15°, and 5.8-fold (95% CI = 2.0–16.8, *p* = 0.001) in the case of A/P_below_ < 0.9, responsible for DCS (Table 4). Another statistic study revealed significant correlation among T1S, T1-T4TK, A/P_below_, A/S + P_below_, and S/I_below_ (Table 5).

**Table 4:** Relative risk ratio in occurrence of spondylolisthesis

	Relative risk	95% CI	*p* value
T1S **>**30°	4.2	2.0–8.9	0.001
T1-T4 TK **>**15°	2.1	1.1–4.3	0.042
A/P_below_ 0.9	5.8	2.0–16.8	0.001

95% CI: 95% confidence interval.

T1S: T1 slope.

TK: thoracic kyphosis angle.

A/P_below_: the ratio of anterior wall height to posterior wall height of the vertebra below seen from the slippage.

**Table 5:** Correlations among sagittal parameters and trapezoidal features.

	T1S	T1-T4 TK	A/P_below_	A/SP_below_	S/I_below_
T1S	1				
T1-T4 TK	0.51	1			
A/P_below_	−0.51	−0.50	1		
A/SP_below_	−0.40	−0.40	0.55	1	
S/I_below_	−0.41	−0.41	0.53	0.99	1

T1S: T1 slope.

TK: thoracic kyphosis angle.

A/P_below_: the ratio of anterior wall height to posterior wall height of the vertebra below seen from the slippage.

A/SP_below_: the ratio of superior end-plate width to sum of superior end-plate width and inferior end-plate width of the vertebra below seen from the slippage.

S/I_below_: the ratio of superior end-plate width to inferior end-plate width of the vertebra below seen from the slippage.

## Discussion

We revealed that DCS was correlated with steep slope in the cervico-thoracic junction region (T1S: 29.9 ± 2.3°), excessive kyphosis at the upper thoracic region (T1-T4TK: 3.5 ± 3.6°), and trapezoidal deformity in the vertebra below the slipped segment (C4-A/P: 0.87 ± 0.02 and C5-A/P: 0.88 ± 0.03) in the current study. These parameters correlated with each other, and might contribute to the pathophysiology of DCS. To the best of our knowledge, morphologic analysis on the sagittal spinal alignment, including trapezoidal deformity in DCS, has not been previously reported. Enlarged T1S, amplified T1-T4TK, and trapezoidal vertebra below the slipped segment were identified as risk factors for DCS in this study.

DCS is a cervical deformity caused by sliding shear force, and disc degeneration, characterized by hypertrophic osteophytes which may act to preserve stability of the affected segment^1,2,11^. In our study, we found evidence of DCS at C2-C3 in 1 patient (3.7%), at C3-C4 in 16 (59%), at C4-C5 in 10 (37%), and at C5-C6 in 1 (3.7%). These findings might be similar to previous reports^1,11,12^. The prevalence of DCS in the lower cervical region was not consistent among reports. There might be some tendency towards higher prevalence of DCS at the lower cervical region in the population of outpatients^1^, and relatively lower prevalence in the population which received operative intervention^11,12^. These findings are perhaps able to rephrase the findings of a previous report which identified two patterns of DCS, the first and more common type of listhesis adjacent to a relatively stiff, spondylotic segment and the second, less common type of listhesis within a spondylotic segment^12^. Our results may tend to favor the prior model mainly due to selection of the operated population. Some previous papers reported that the middle cervical region was loaded with tremendous mechanical stress, which militated in a sliding direction, in the course of regressive changes whereas the lower cervical spine change their morphology and lost the flexibility and elasticity^20,21^, which is considered as the preventive factors against listhesis in lower cervical region. Due to these regressive conditions, which seem similar to the pathophysiology of adjacent segmental disease above the fused spinal segment^22^, hypermobility of the middle cervical region above the trapezoidal and stiffened lower cervical region assumes DCS as a result. This tentative hypothesis seems to support our results presenting relatively low prevalence of DCS at the lower cervical region with some trapezoidal deformity, which represents the regressive change, and high prevalence of DCS at the middle cervical region above the vertebrae with trapezoidal features.

For the findings that T1S and T1-T4TK exhibited significant correlation with DCS, both logistic and linear regression models were performed. These analyses revealed the thresholds of approximate 30° for T1S and 15° for T1-T4TK responsible for occurrence of DCS defined by the amount of slippage >2 mm. It is difficult to interpret these findings as results or causes, because this study is a cross-sectional analysis. Perhaps it helps to interpret these findings as that T1S has been reported responsible for the contour above^23^ and that enlarged T1S, which is representative of kyphotic T1-T4TK and vice versa, produces sliding shear force for the contour above which has the trapezoidal feature, and under these assumptions DCS may occur. For the findings of high relative risk of cervical positive imbalance arising from T1S >30°, T1S may be an important parameter regarding the pathophysiology of DCS and the supervenient local kyphosis, where neurological function has possibly worsened^24^.

One strength of the current study is the inclusion of a conspecific population of consecutive DCS patients who have trapezoidal deformity in the segment below. Limitations to this study must be acknowledged also. First, the population is small and has some skewed characteristics arising from inclusion and exclusion criteria. Second, we did not analyze the symptoms or the health-related quality of life scores of affected individuals. Despite these limitations, we could consider this study to have some importance towards understanding the pathophysiology of DCS. Further studies are needed from a larger sample size of patients including a heterogeneous population. To clarify the clinical significance, such as the correlation between the amount of DCS and the clinical results or the effect of DCS on clinical symptoms in a grade-wise manner, further study is also needed in a systematic way, including both asymptomatic patients and symptomatic patients.

## Conclusion

DCS was mostly found in the middle cervical region. Among sagittal parameters, enlarged T1S and T1-T4TK, which were strongly correlated with amount of slippage, was considered affected to DCS. Cervical kyphosis and trapezoidal deformity also exhibited strong correlations with DCS, and were considered responsible for clinical instability.
